# The impact of physical activity on fatigue and quality of life in lung cancer patients: a randomised controlled trial protocol

**DOI:** 10.1186/1471-2407-12-572

**Published:** 2012-12-05

**Authors:** Haryana M Dhillon, Hidde P van der Ploeg, Melanie L Bell, Michael Boyer, Stephen Clarke, Janette Vardy

**Affiliations:** 1 Centre for Medical Psychology and Evidence-based Decision-making, University of Sydney, Sydney, Australia; 2 Sydney School of Public Health, University of Sydney, Sydney, Australia; 3 Department of Public and Occupational Health, VU University Medical Center Amsterdam, Amsterdam, The Netherlands; 4 Psycho-Oncology Co-Operative Research Group (PoCoG), School of Psychology, Faculty of Science, University of Sydney, Sydney, Australia; 5 Sydney Cancer Centre, Sydney, Australia; 6 Sydney Medical School, University of Sydney, Sydney, Australia; 7 Royal North Shore Hospital, St Leonards, Australia; 8 Sydney Cancer Centre, Concord Repatriation General Hospital, Hospital Rd, Concord, NSW, 2137, Australia

**Keywords:** Physical activity, Exercise, Fatigue, Quality of life, Lung cancer

## Abstract

**Background:**

People with lung cancer have substantial symptom burden and more unmet needs than the general cancer population. Physical activity (PA) has been shown to positively influence quality of life (QOL), fatigue and daily functioning in the curative treatment of people with breast and colorectal cancers and lung diseases, as well as in palliative settings. A randomised controlled trial (RCT) is needed to determine if lung cancer patients benefit from structured PA intervention. The Physical Activity in Lung Cancer (PAL) trial is designed to evaluate the impact of a 2-month PA intervention on fatigue and QOL in patients with non-resectable lung cancer. Biological mechanisms will also be studied.

**Methods/design:**

A multi-centre RCT with patients randomised to usual care or a 2-month PA programme, involving supervised PA sessions including a behavioural change component and home-based PA. QOL questionnaires, disease and functional status and body composition will be assessed at baseline, 2, 4 and 6 months follow-up. The primary endpoint is comparative levels of fatigue between the 2 arms. Secondary endpoints include: QOL, functional abilities and physical function. Exploratory endpoints include: anxiety, depression, distress, dyspnoea, PA behaviour, fitness, hospitalisations, survival, cytokines and insulin-like growth factor levels.

**Discussion:**

This study will provide high-level evidence of the effect of PA programmes on cancer-related fatigue and QOL in patients with advanced lung cancer. If positive, the study has the potential to change care for people with cancer using a simple, inexpensive intervention to improve their QOL and help them maintain independent function for as long as possible.

**Trial registration:**

Australian New Zealand Clinical Trials Registry No. ACTRN12609000971235

## Background

Lung cancer is the leading cause of cancer-related death worldwide. In the United States alone it was estimated there would be 226,160 new lung cancer cases and 160,340 lung cancer deaths in 2012 [1]. The majority (60%) of lung cancer patients present with advanced, incurable disease resulting in a median survival of only 9–12 months [2]. In this context, the aim of anti-cancer treatment is to improve or maintain quality of life (QOL) in addition to prolonging survival.

Fatigue is one of the most common and distressing patient-reported symptoms associated with cancer and its treatment [3-5]. People with lung cancer report a higher prevalence and longer duration of cancer-related fatigue, leading to more functional impairment when compared to other cancer patients [6]. There is growing evidence that physical activity can improve fatigue in people with cancer, [7-9] and in those with chronic obstructive pulmonary disease (COPD) [10]. However, no study has evaluated the benefits of a lifestyle physical activity programme for people with non-resectable lung cancer. This study will determine if physical activity improves fatigue, in people with non-resectable lung cancer.

In the past five years several large observational studies have reported that physical activity reduces the risk of cancer recurrence, death from cancer, and death from all causes for breast and colon cancer patients [11-16]. These studies have shown consistently that more than 9 metabolic equivalent task (MET) hours/week of physical activity (e.g. walking at a moderate pace for 2.5 hours/week) is associated with improved health outcomes. This is similar to the health guidelines of 150 minutes of moderate intensity physical activity per week or 60 minutes of vigorous physical activity per week [17].

A burgeoning literature has examined the effects of physical activity on supportive care outcomes in people with cancer including physical fitness, physical function, fatigue and QOL [8,18-21]. Observed benefits of physical activity include improved cardiovascular and pulmonary function, musculoskeletal strength, maintenance of mobility and independence, and improved psychological well-being and QOL [9,22,23]. Systematic reviews and meta-analyses have concluded that physical activity interventions during and after cancer therapies often result in meaningful and reliable improvements in several supportive care outcomes [8,9,19,20,22-25]. These benefits include observed changes in physiologic measures, objective performance indicators, self-reported functioning and symptoms, psychological well-being and overall QOL. However, these studies did not include people with advanced lung cancer.

A large body of evidence documents the benefits of physical activity as a component of pulmonary and cardiac rehabilitation in similar clinical populations, which demonstrate improved QOL, functional status, and symptom control including dyspnoea in patients with COPD. A Cochrane review of 31 randomised controlled trials in chronic obstructive pulmonary disease patients found those receiving pulmonary rehabilitation had statistically and clinically significant improvements in QOL, including dyspnoea, fatigue and patient control over disease [10].

Despite the benefits of physical activity, only 20-32% of all cancer survivors report meeting physical activity guidelines [18,26-28]. A recent study in lung cancer survivors found that 73% did not meet the United States physical activity guidelines, and 51% did not participate in any moderate or vigorous physical activity at all [29]. Lung cancer survivors who met the physical activity guidelines reported significantly better QOL across multiple domains compared to those who did not meet the guidelines [30]. This suggests that there are considerable opportunities for improving the physical activity behaviour of lung cancer patients and possibly improving their QOL.

Most physical activity studies have been performed in cancer populations with potentially curative disease, particularly breast and colorectal cancer survivors. By comparison, people with advanced incurable lung cancer are older (median age of 70 years) and more likely to have co-morbidities such as chronic obstructive pulmonary disease and cardiovascular disease. However, physical activity has been found to be beneficial in older cancer patients, [3] and in a palliative population [21]. Cardiopulmonary exercise testing in people with advanced cancer, including lung cancer, has been shown to be safe and feasible, which suggests that most people would be able to follow a programme emphasising moderate intensity physical activity [31].

The biological mechanisms by which physical activity may modify cancer risk or disease progression, or improve symptom control, include changes in insulin-like growth factor (IGF) levels, immune regulation, sex and metabolic hormone levels, prostaglandin ratio and obesity [16,32-35]. More specific to lung cancer, physical activity improves pulmonary function and perfusion, [36] and it is hypothesised that physical activity may up-regulate antioxidants and free scavengers to help counteract the effects of cigarette smoke [37]. Physical activity may also decrease the risk of pneumonia and venothrombotic events, thereby improving overall survival and QOL in patients with non-resectable lung cancer.

Our aim is to evaluate a physical activity programme in people with non-resectable lung cancer to determine if it improves fatigue and quality of life. Our hypotheses are that people who undergo the physical activity intervention compared to those who do not, will: 1) report less fatigue; 2) have better health-related quality of life; 3) have improved overall survival; and, 4) have less decline in their level of physical function over time.

## Methods/design

This multi-site, randomised controlled trial is being led by the Survivorship Research Group (SuRG) at the University of Sydney, Australia. Funding was obtained from a Young Investigator Award from the Lance Armstrong Foundation. The study was prospectively registered with the Australian New Zealand Clinical Trials Registry, registration number ACTRN12609000971235. Ethics approval has been obtained from Concord Repatriation General Hospital Human Research Ethics Committee for each participating institution under the New South Wales Health multi-site ethics approval scheme (HREC reference number: 08/CRGH/242).

Participants are recruited from lung cancer clinics at 4 Sydney hospitals, commencing May 2009 until achievement of planned sample size.

**Figure 1:**
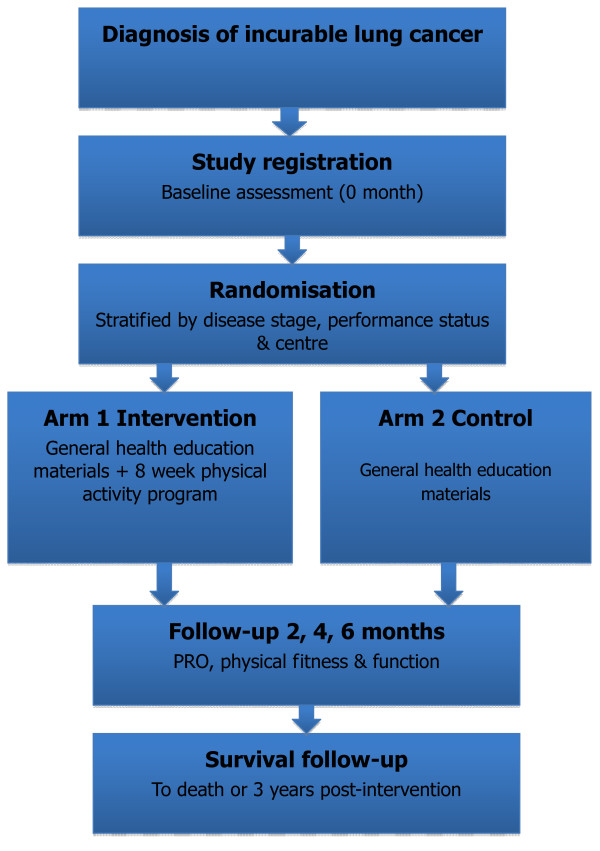
**Study flowchart.** PRO = patient reported outcomes.

Consenting, medically fit people with non-resectable lung cancer, including non-small cell lung cancer (NSCLC) and small cell lung cancer (SCLC) are randomised to usual care or a 2-month physical activity intervention (Figure 1). People with Stage III NSCLC or locally advanced SCLC being treated with curative intent must have completed combination chemo-radiotherapy a minimum of 4 weeks prior to randomisation, and have an incomplete response on staging CT scans. People with metastatic disease being treated palliatively may be receiving chemotherapy, radiotherapy and/or biological agents. All subjects must be medically fit to participate in a physical activity program, as assessed by the Physical Activity Readiness Questionnaire (PAR-Q) [38] and their treating physician. Major exclusion criteria are: Eastern Co-operative Oncology Group (ECOG) performance status ≥3, life expectancy < 6 months or insufficient English fluency to complete the questionnaires. Complete inclusion and exclusion criteria are outlined in Table 1. Randomisation is stratified for disease stage (locally advanced: NSCLC Stage III/ limited SCLC without a complete response to treatment versus metastatic disease: NSCLC Stage IV and extensive SCLC), ECOG performance status (0–1 versus 2), and cancer centre. It is performed by an external academic organisation using an interactive voice response system.

**Table 1:** Study inclusion and exclusion criteria

**Inclusion criteria**	**Exclusion criteria**
Diagnosis of invasive lung cancer (NSCLC or SCLC) that, in the opinion of the oncology team, is non-resectable and non-curable.	European Co-operative Oncology Group (ECOG) Performance Status of > 3.
People with Stage III NSCLC or limited SCLC who have evidence of residual disease and have completed treatment with chemotherapy and or radiotherapy a minimum of 4 weeks prior to commencing the study.	Pre-existing significant co-morbid conditions precluding participation in a physical activity programme, as determined by the investigator.
People with advanced disease being treated palliatively may be receiving chemotherapy, biological agents and/or best supportive care.	Insufficient English fluency to complete the questionnaires.
Aged at least 18 years.	Life expectancy of < 6 months.
Medically fit to participate in a physical activity programme, as determined by their oncologist.	Inability to complete the baseline exercise test (6 Minute Walk) done prior to randomisation.
Ability (i.e. sufficiently fluent) and willingness to complete the patient- reported outcome questionnaires, physical activity questionnaires and logs in English.	
Give written informed consent.	
Completion of the 3 Questionnaire assessment within 14 days of registration.	
Completion of the Physical Activity Readiness Questionnaire within 14 days of registration.	

*NSCLC*, Non-small cell lung cancer.

*SCLC*, Small cell lung cancer.

### Control – standard of care

All participants will have routine follow-up as per local cancer centre practice and receive cancer-specific education materials regarding nutrition (Eat For Health) and exercise (Move Your Body) published by Cancer Council Australia (http://www.cancer.org.au/home.htm) to encourage adoption of a healthy lifestyle.

### Intervention

#### Theoretical framework

The physical activity programme is based on the Theory of Planned Behaviour, [39] and modelled after the successful behaviour change programme developed by the Diabetes Prevention Programme [40].

Participants randomised to the physical activity intervention arm will participate in a 2-month individualised physical activity and behavioural support programme with a Physical Activity Consultant (PAC).

The goal of the intervention will be to increase the participant’s physical activity over the 2-month intervention by a minimum of 3 MET hours per week compared to baseline. While this may be less than the recommended physical activity guidelines for some participants, those capable of increasing their MET hours further will be encouraged to do so.

The physical activity program will consist of supervised physical activity (30 to 45 minutes duration) and behaviour support sessions (15 to 30 minutes) once a week for 8 weeks, as well as unsupervised home physical activity sessions. The physical activity program (including supervised and unsupervised sessions) will be tailored to the participant’s baseline fitness, performance status and physical activity preferences, and take account of personal facilitators and barriers to physical activity. The study emphasises aerobic physical activity. Consequently it is expected that most participants will engage in walking as their preferred form of physical activity; however, other forms of aerobic activity such as swimming, cycling or running are acceptable. The program also incorporates advice about resistance exercises to encourage participants to maintain muscle function and strength.

The weekly structured sessions include physical activity as well as behaviour support sessions that use behaviour lifestyle change principles. It is the behaviour support sessions that will be the primary focus of the program promoting unsupervised physical activity. A full list of behaviour change session titles and their learning goals is outlined in Table 2. Participants will be supplied with a physical activity and behaviour change guidebook titled “Exercising with Lung Cancer”, a pedometer and physical activity diary for use throughout the intervention.

**Table 2:** Behaviour support sessions

Session*	**Session title**	**Session learning goals**
		**By the end of the session participants should have**:
1	Introduction to the program	· Learned about the gymnasium / fitness facility;
		· Learned how to monitor intensity using the Borg Scale;
		· Learned how to properly stretch;
		· Learned about the PAL Study objectives;
		· Learned how to complete the daily PA Diary.
2	Exercising properly	· Learned what to wear when doing PA;
		· Learned the basics of proper hydration;
		· Learned about safety when doing PA.
3	Goal setting and planning	· Understood the importance of setting goals;
		· Learned how to create short and long term goals based on the SMART principle;
		· Learned how to create action steps to achieve a PA goal.
4	Pedometers and walking	· Learned about the health benefits of walking;
		· Learned how to use a pedometer;
		· Create a plan to increase the amount of steps per day.
5	Benefits of physical activity and overcoming barriers to physical activity	· Understood the benefits of PA to the general population;
		· Learned more about the benefits of PA specific to people with lung cancer;
		· Identified what their personal barriers to PA are;
		· Worked through and brainstormed ideas how to overcome these barriers.
6	Environmental scan	· Learned what PA opportunities exist in their local environment;
		· Learned how to make use of indoor fitness facilities or sports stores.
7	Social support and having fun with physical activity	· Learned the importance of social support for maintaining a PA program;
		· Identified what social support they may be able to utilise;
		· Identified what makes PA fun for them;
		· Brainstormed how to increase their enjoyment of PA.
8	Stimulus control and decision balance sheet	· Learned what stimulus control is and how it affects behaviour;
		· Learned how to establish appropriate stimuli;
		· Understood the benefits of using a decision balance sheet;
		· Learned how to create and use a decision balance sheet for themselves.

* Order of behaviour change session delivery can be tailored to suit the most pressing educational and behavioural needs of participants.

### Assessments

Outcomes will be assessed at baseline (prior to randomisation), 2, 4 and 6 months (Figure 1). Baseline assessments involve a medical history and examination. At all assessments, disease and treatment status will be recorded and subjects will complete patient reported outcome (PRO) questionnaires, a standardised fitness test (6 Minute Walk Test, [41] Senior’s Fitness Test, [41] hand grip strength [42]), and donate blood for biomarker analysis. All subjects will wear an Actigraph GT1M accelerometer [43] (Actigraph LLC, Pensacola, FL, USA) on the right hip to objectively determine physical activity for 7 full days prior to each assessment. Survival status will be obtained from the medical records, with follow up until the time of the final analysis.

#### Primary and secondary endpoints

The primary endpoint will be the level of self-reported fatigue as assessed by the Functional Assessment of Cancer Therapy - fatigue (FACT-F) subscale [44,45]. The primary outcome will be a comparison of fatigue in the intervention and control arms at the conclusion of the intervention (i.e. at the 2-month assessment).

Secondary endpoints will include the following Patient Reported Outcomes:

• QOL (European Organisation for Research and Treatment of Cancer –Quality of life questionnaire – Core (EORTC-QLQ-C30) and Lung module (LC-13 subscale) [46],

• activities of daily living and instrumental activities of daily living[47].

• physical function (6 Minute Walk Test) [41].

Other exploratory endpoints relating to physical activity and fitness will include:

• anxiety and depression (General Health Questionnaire 12) [48];

• distress (Distress Thermometer) [49],

• perceived cognitive function (FACT-Cognition v3) [50],

• sleep quality (Pittsburgh Sleep Quality Index) [51],

• dyspnoea (University of California San Diego Shortness of Breath Questionnaire [52] and physical activity and sedentary behaviour (Actigraph GT1M accelerometer; Active Australia questionnaire [53], sedentary behaviour questionnaire [54]);

• physical activity attitudes (Social Cognitive Determinants of Exercise questionnaire) [30,55]

• physical fitness (6 Minute Walk Test, Seniors Fitness Test, [41] hand grip strength);

• pulmonary function (Forced Expiratory Volume in 1 second, Forced Vital Capacity);

• general health, functional and performance status (anthropmetric measurements, Colinet co-morbidity score [56], ECOG performance status, admissions to hospital/hospice and survival data); and

• mechanisms and prognostic biomarkers (Glasgow Prognostic Score [C-reactive protein, albumin], selected cytokine levels, insulin-like growth factors).

Table 3 gives an overview of all outcome assessments.

**Table 3:** Assessments

	**Investigations**	**Timing**
History and Physical Exam including:	· Height (baseline only)	0,2,4, 6 months
	· Weight and Body Mass Index (BMI)	
	· Blood Pressure and Heart Rate	
	· Oxygen saturation (SaO2) (on room air)	
	· Concomitant medications (any changes to baseline meds)	
	· Colinet Co-morbidity Score	
	· Eastern Cooperative Oncology Group Performance Status (ECOG PS)	
	· Disease status	
	· Current treatments	
Fitness Testing	· 6-minute walk test (6MWT)	0,2,4, 6,months
	· Senior’s Fitness Test	
	· Hand grip strength	
Pulmonary Function	· Forced Expiratory Volume at 1 second (FEV_1_)	0,2,4,6 months
	· Forced Vital Capacity (FVC)	
Adverse Events	· Adverse events will be recorded and graded using National Cancer Institute Common Terminology Criteria for Adverse Events Version 3.0 (NCI CTCAE v3.0)	0,2,4, 6 months
Patient Reported Outcomes	· Functional Assessment of Cancer Therapy - Fatigue (FACT-F subscale)	0,2,4,6 months
	· Quality of Life (QOL) European Organisation for Research and Treatment of Cancer –Quality of life questionnaire – Core (EORTC-QLQ-C30) and Lung module (LC-13 subscale)	
	· Depression and Anxiety (General Health Questionnaire - GHQ12)	
	· Sleep Quality (Pittsburgh Sleep Quality Index - PSQI)	
	· Cognitive Function (Functional Assessment of Cancer Therapy – Cognitive scale - FACT-COG v3)	
	· Distress (Distress Thermometer)	
	· Dyspnoea (The University of California, San Diego Shortness of Breath Questionnaire -SOBQ)	
	· Activities of Daily Living (ADLs) and Independent Activities of Daily Living (IADLs)	
	· Sedentary time (Sitting Questionnaire)	
	· Physical activity attitudes (Social Cognitive Determinants of Exercise questionnaire)	
Physical Activity Behaviour/ Adherence	· Physical activity participation (Active Australia)	0,2,4, 6 months
	· Accelerometer -for one week prior to each assessment	
Other Investigations	· Fasting Serum/plasma collection for correlative studies and optional banking	0,2,4,6 months
	· Glasgow Prognostic score: Full blood count (FBC), Albumin, C-reactive protein (CRP)	

### Statistical analysis

#### Sample size

A sample size of 72 evaluable participants (36/arm) provides 80% power (two-tailed α 0.05) to detect a difference of 6 points (FACT-F subscale range: 0–52) in the primary outcome measure of fatigue between the two treatment arms at 2 months and assuming a standard deviation of 9 [57]. This is a standardized effect size of 0.67, thus each of the secondary outcomes is also powered at this level. Due to the poor prognosis for this patient population we need to increase our enrolment by 30% to account for attrition. Therefore, in total we will aim to recruit ~102 patients.

#### Statistical analyses

We report only on analyses for the primary and three secondary outcomes: fatigue, quality of life, activities of daily living, and the six minute walk test. Because of the potential for informative dropout, rigorous methods which account for missing data will be employed. There are three type of missing data. Data are missing completely at random (MCAR) if missing values and observed value are not systematically different. Data are missing at random (MAR) if any systematic differences can be explained by observed outcome or covariate data. Data are missing not at random (MNAR) when systematic differences exist, even after adjusting for previously observed data. A MAR primary analysis followed by sensitivity analyses are recommended by experts in the field of missing data [58].

Thus, our main analyses will use linear mixed models for all outcomes, which are unbiased for data which are MCAR or MAR and are consistent with the intention-to-treat principle [58-60]. Mixed model account for covariance between repeated measures on patients and allow for: 1) comparing patterns of change over time by testing the intervention group by time interaction; and 2) estimating and testing differences between groups at time points of interest via linear contrasts. Unadjusted and adjusted main analyses will be carried out, with adjustment for baseline measures including sex, age, disease stage, and the baseline outcome.

Patterns of missing data will be assessed to consider potential missing data mechanisms and baseline characteristics of patients lost to follow-up at 2, 4 and 6 months will be compared to patients who completed follow-up to assess patterns of loss to follow-up. Sensitivity analyses will be carried out using multiple imputation, which will include auxiliary data such as ECOG performance status and baseline secondary outcomes such as physical activity measures.

Overall survival is an exploratory endpoint, because the study has limited power to evaluate this. Patients alive at final analysis or who have become lost to follow up will be censored at their last contact date. Overall survival will be described by the Kaplan-Meier method. A stratified log-rank test adjusting for the stratification factors of disease stage (locally advanced vs metastatic) and ECOG performance status (0+1 vs 2) at randomisation will be used to compare overall survival between the two arms.

## Discussion

Advanced lung cancer is incurable, and is the leading cause of cancer deaths world-wide. The goal of treatment is to improve QOL, maintain physical function and prolong life. Treatments to date have focused on disease modifying anti-cancer therapies (e.g. chemotherapy or radiation). A focus on the person and their functional status reflects an innovative approach to therapy, maximising their ability to live with miminal effects of the disease for as long as possible.

A physically active lifestyle improves fatigue and QOL in other cancer populations, particularly in early stage disease, but this type of intervention has not been evaluated in advanced lung cancer, where people are generally older with a higher burden of co-morbid illnesses than other cancer groups. This intervention has the potential to change standard care with a simple, safe, relatively inexpensive treatment that could improve the QOL of people with lung cancer and help them to maintain independent function for as long as possible. This study will determine whether a physical activity programme reduces the number of days participants spend in hospital for symptom control and end of life care. It will obtain unique data about the impact of physical activity on cytokines and the insulin pathway and the influence of each of these biomarkers on prognosis. It will provide preliminary evidence on whether physical activity can impact on survival in people with lung cancer.

One of the greatest challenges to implementation of physical activity programmes has been availability of adequate support and resources to promote and maintain adherence with physical activity. If a randomised controlled trial demonstrated a significant benefit for people with lung cancer this would not only provide great impetus to patients to increase their physical activity and oncologists to promote it, but provide an evidence base to support a change in policy and practice to ensure patients engage with a physical activity consultant to design and follow-up a personalised physical activity programme. The intervention under study has been designed to ensure it can be offered in a hospital or community setting.

## Conclusions

Physical activity has been associated with many health benefits. This study will provide high-level evidence of the effectiveness of physical activity programmes to improve cancer fatigue and QOL in a cancer population with substantial symptom burden and high unmet needs. It will determine the feasibility of delivering exercise programmes in an advanced lung cancer population, and be powered to evaluate whether physical activity improves fatigue and QOL in this patient population. In addition, it will obtain important data about the impact of physical activity on physical function, body composition, mood, perceived cognitive function, dyspnoea, cytokines and the insulin pathway, and their influence on prognosis. The study outcomes have the potential to change standard care, with a non-toxic and inexpensive treatment, which can improve quality of life of people with lung cancer and help them achieve the highest possible level of independent function for as long as possible [61].

## Competing interests

None of the authors has a conflict of interest to declare.

## Authors’ contributions

HD and JV devised the study concept and design. All authors contributed to the study protocol. M.Bell was responsible for overseeing the statistical section. HD, HvP, M.Bell and JV wrote the manuscript. All authors read and approved the final manuscript.

## Pre-publication history

The pre-publication history for this paper can be accessed here:

http://www.biomedcentral.com/1471-2407/12/572/prepub

## References

[B1] Society AC Cancer facts & figures 2012 2012 Atlanta: American Cancer Society

[B2] Gloeckler LA Eisner MP Ries LAG, Young JL, Keel GE, Eisner MP, Lin YD, Horner M-J Cancer of the lung SEER Survival Monograph: Cancer Survival Among Adults: US SEER Program, 1988–2001, Patient and Tumor Characteristics. Volume NIH Pub. No. 07–6215 2007 Bethesda, MD: National Cancer Institute, SEER Program

[B3] Luctkar-Flude MF Groll DL Tranmer JE Woodend K Fatigue and physical activity in older adults with cancer: a systematic review of the literature Cancer Nurs 2007 30 5 E35 45 10.1097/01.NCC.0000290815.99323.75 17876176

[B4] Joyce M Schwartz S Huhmann M Supportive care in lung cancer Semin Oncol Nurs 2008 24 1 57 67 10.1016/j.soncn.2007.11.013 18222153

[B5] Hofman M Ryan JL Figueroa-Moseley CD Jean-Pierre P Morrow GR Cancer-related fatigue: the scale of the problem Oncologist 2007 12 Suppl 1 4 10 17573451 10.1634/theoncologist.12-S1-4

[B6] Forlenza MJ Hall P Lichtenstein P Evengard B Sullivan PF Epidemiology of cancer-related fatigue in the Swedish twin registry Cancer 2005 104 9 2022 2031 10.1002/cncr.21373 16206253

[B7] Dimeo F Schwartz S Wesel N Voigt A Thiel E Effects of an endurance and resistance exercise program on persistent cancer-related fatigue after treatment Ann Oncol 2008 19 1495 1499 10.1093/annonc/mdn068 18381369

[B8] Galvao DA Newton RU Review of exercise intervention studies in cancer patients J Clin Oncol 2005 23 4 899 909 10.1200/JCO.2005.06.085 15681536

[B9] Stevinson C Lawlor DA Fox KR Exercise interventions for cancer patients: systematic review of controlled trials Cancer Causes Control 2004 15 10 1035 1056 10.1007/s10552-004-1325-4 15801488

[B10] Lacasse Y Goldstein R Lasserson TJ Martin S Pulmonary rehabilitation for chronic obstructive pulmonary disease Cochrane Database Syst Rev 2006 4 CD003793 17054186 10.1002/14651858.CD003793.pub2

[B11] Holmes MD Chen WY Feskanich D Kroenke CH Colditz GA Physical activity and survival after breast cancer diagnosis JAMA 2005 293 20 2479 2486 10.1001/jama.293.20.2479 15914748

[B12] Meyerhardt JA Giovannucci EL Holmes MD Chan AT Chan JA Colditz GA Fuchs CS Physical activity and survival after colorectal cancer diagnosis J Clin Oncol 2006 24 22 3527 3534 10.1200/JCO.2006.06.0855 16822844

[B13] Meyerhardt JA Heseltine D Niedzwiecki D Hollis D Saltz LB Mayer RJ Thomas J Nelson H Whittom R Hantel A Impact of physical activity on cancer recurrence and survival in patients with stage III colon cancer: findings from CALGB 89803 J Clin Oncol 2006 24 22 3535 3541 10.1200/JCO.2006.06.0863 16822843

[B14] Haydon AM Macinnis RJ English DR Giles GG Effect of physical activity and body size on survival after diagnosis with colorectal cancer Gut 2006 55 1 62 67 10.1136/gut.2005.068189 15972299 PMC1856365

[B15] Meyerhardt JA Giovannucci EL Ogino S Kirkner GJ Chan AT Willett W Fuchs CS Physical activity and male colorectal cancer survival Arch Intern Med 2009 169 22 2102 2108 10.1001/archinternmed.2009.412 20008694 PMC2852183

[B16] Ballard-Barbash R Friedenreich CM Courneya KS Siddiqi SM McTiernan A Alfano CM Physical activity, biomarkers, and disease outcomes in cancer survivors: a systematic review J Natl Cancer Inst 2012 104 815 840 10.1093/jnci/djs207 22570317 PMC3465697

[B17] World Health Organisation Global recommendations on physical activity for health 2010 Geneva, Switzerland: World Health Organization 26180873

[B18] Lynch BM Cerin E Owen N Aitken JF Associations of leisure-time physical activity with quality of life in a large, population-based sample of colorectal cancer survivors Cancer Causes Control 2007 18 7 735 742 10.1007/s10552-007-9016-6 17520334

[B19] Courneya KS Friedenreich CM Quinney HA Fields AL Jones LW Fairey AS A randomized trial of exercise and quality of life in colorectal cancer survivors Eur J Cancer Care 2003 12 4 347 357 10.1046/j.1365-2354.2003.00437.x 14982314

[B20] Knols R Aaronson NK Uebelhart D Fransen J Aufdemkampe G Physical exercise in cancer patients during and after medical treatment: a systematic review of randomized and controlled clinical trials J Clin Oncol 2005 23 16 3830 3842 10.1200/JCO.2005.02.148 15923576

[B21] Oldervoll LM Loge JH Paltiel H Asp MB Vidvei U Wiken AN Hjermstad MJ Kaasa S The effect of a physical exercise program in palliative care: a phase II study J Pain Symptom Manage 2006 31 5 421 430 10.1016/j.jpainsymman.2005.10.004 16716872

[B22] Wiggins MS Simonavice EM Cancer prevention, aerobic capacity, and physical functioning in survivors related to physical activity: a recent review Cancer Manage Res 2010 2 157 164 10.2147/cmar.s7461 PMC3004575 21188106

[B23] Speck RM Courneya KS Masse LC Duval S Schmitz KH An update of controlled physical activity trials in cancer survivors: a systematic review and meta-analysis J Cancer Surviv: Res and Pract 2010 4 2 87 100 10.1007/s11764-009-0110-5 20052559

[B24] Schmitz KH Holtzman J Courneya KS Masse LC Duval S Kane R Controlled physical activity trials in cancer survivors: a systematic review and meta-analysis Cancer Epidemiol Biomarkers Prev 2005 14 7 1588 1595 10.1158/1055-9965.EPI-04-0703 16030088

[B25] Schmitz KH Courneya KS Matthews C Demark-Wahnefried W Galvao DA Pinto BM Irwin ML Wolin KY Segal RJ Lucia A American College of Sports Medicine roundtable on exercise guidelines for cancer survivors Med Sci in Sports Exerc 2010 42 7 1409 1426 10.1249/MSS.0b013e3181e0c112 20559064

[B26] Bellizzi KM Rowland JH Jeffery DD McNeel T Health behaviors of cancer survivors: examining opportunities for cancer control intervention J Clin Oncol 2005 23 34 8884 8893 10.1200/JCO.2005.02.2343 16314649

[B27] Courneya KS Katzmarzyk P Bacon E Physical activity and obesity in canadian cancer survivors: population-based estimates from the 2005 canadian community health survey Cancer 2008 112 11 2475 82 10.1002/cncr.23455 18428195

[B28] Coups EJ Ostroff JS A population-based estimate of the prevalence of behavioral risk factors among adult cancer survivors and noncancer controls Prev Med 2005 40 6 702 711 10.1016/j.ypmed.2004.09.011 15850868

[B29] Oh B Butow P Mullan B Clarke S Beale P Pavlakis N Kothe E Lam L Rosenthal D Impact of medical Qigong on quality of life, fatigue, mood and inflammation in cancer patients: a randomized controlled trial Ann Oncol 2012 21 3 608 614 19880433 10.1093/annonc/mdp479 PMC2826100

[B30] Azjen I The theory of planned behavior Organ Behav Hum Decis Process 1991 50 179 211 10.1016/0749-5978(91)90020-T

[B31] Jones LW Eves ND Mackey JR Peddle CJ Haykowsky M Joy AA Courneya KS Tankel K Spratlin J Reiman T Safety and feasibility of cardiopulmonary exercise testing in patients with advanced cancer Lung cancer (Amsterdam, Netherlands) 2007 55 2 225 232 10.1016/j.lungcan.2006.10.006 17113185

[B32] McTiernan A Mechanisms linking physical activity with cancer Nat Rev 2008 8 3 205 211 10.1038/nrc2325 18235448

[B33] Friedenreich CM Orenstein MR Physical activity and cancer prevention: etiologic evidence and biological mechanisms J Nutr 2002 132 11 Suppl 3456S 3464S 12421870 10.1093/jn/132.11.3456S

[B34] Fairey AS Courneya KS Field CJ Bell GJ Jones LW Mackey JR Effects of exercise training on fasting insulin, insulin resistance, insulin-like growth factors, and insulin-like growth factor binding proteins in postmenopausal breast cancer survivors: a randomized controlled trial Cancer Epidemiol Biomarkers Prev 2003 12 8 721 727 12917202

[B35] Irwin ML Varma K Alvarez-Reeves M Cadmus L Wiley A Chung GG Dipietro L Mayne ST Yu H Randomized controlled trial of aerobic exercise on insulin and insulin-like growth factors in breast cancer survivors: the yale exercise and survivorship study Cancer Epidemiol Biomarkers Prev 2009 18 1 306 313 10.1158/1055-9965.EPI-08-0531 19124513 PMC2841479

[B36] Jakes RW Day NE Patel B Khaw KT Oakes S Luben R Welch A Bingham S Wareham NJ Physical inactivity is associated with lower forced expiratory volume in 1 second: European prospective investigation into cancer-Norfolk prospective population study Am J Epidemiol 2002 156 2 139 147 10.1093/aje/kwf021 12117705

[B37] Morrow JD Frei B Longmire AW Gaziano JM Lynch SM Shyr Y Strauss WE Oates JA Roberts LJ 2nd Increase in circulating products of lipid peroxidation (F2-isoprostanes) in smokers. Smoking as a cause of oxidative damage N Engl J Med 1995 332 18 1198 1203 10.1056/NEJM199505043321804 7700313

[B38] Thomas S Reading J Shephard RJ Revision of the physical activity readiness questionnaire (PAR-Q) Can J Sport Sci = Journal canadien des sciences du sport 1992 17 4 338 345 1330274

[B39] Ajzen I The theory of planned behavior Organ Behav Hum Decis Process 1991 50 179 211 10.1016/0749-5978(91)90020-T

[B40] Knowler WC Barrett-Connor E Fowler SE Hamman RF Lachin JM Walker EA Nathan DM Reduction in the incidence of type 2 diabetes with lifestyle intervention or metformin N Engl J Med 2002 346 6 393 403 11832527 10.1056/NEJMoa012512 PMC1370926

[B41] Rikli R Jones CJ Senior fitness test manual 2001 Champaign, IL: Human Kinetics

[B42] Bohannon RW Hand-grip dynamometry provides a valid indication of upper extremity strength impairment in home care patients J Hand Ther: official journal of the American Society of Hand Therapists 1998 11 4 258 260 10.1016/S0894-1130(98)80021-5 9862263

[B43] Plasqui G Westerterp KR Physical activity assessment with accelerometers: an evaluation against doubly labeled water Obes (Silver Spring) 2007 15 10 2371 2379 10.1038/oby.2007.281 17925461

[B44] Yellen SB Cella DF Webster K Blendowski C Kaplan E Measuring fatigue and other anemia-related symptoms with the Functional Assessment of Cancer Therapy (FACT) measurement system J Pain Symptom Manage 1997 13 2 63 74 10.1016/S0885-3924(96)00274-6 9095563

[B45] Cella D Davis K Breitbart W Curt G Cancer-related fatigue: prevalence of proposed diagnostic criteria in a United States sample of cancer survivors J Clin Oncol 2001 19 14 3385 3391 11454886 10.1200/JCO.2001.19.14.3385

[B46] Bergman B Aaronson NK Ahmedzai S Kaasa S Sullivan M The EORTC QLQ-LC13: a modular supplement to the EORTC core quality of life questionnaire (QLQ-C30) for use in lung cancer clinical trials. EORTC study group on quality of life Eur J Cancer 1994 30A 5 635 642 8080679 10.1016/0959-8049(94)90535-5

[B47] Thomas VS Rockwood K McDowell I Multidimensionality in instrumental and basic activities of daily living J Clin Epidemiol 1998 51 4 315 321 10.1016/S0895-4356(97)00292-8 9539888

[B48] Goldberg DP A user's guide to the general health questionnaire 1991 Windsor, UK: NFER-Nelson

[B49] Tuinman MA Gazendam-Donofrio SM Hoekstra-Weebers JE Screening and referral for psychosocial distress in oncologic practice: use of the distress thermometer Cancer 2008 113 4 870 878 10.1002/cncr.23622 18618581

[B50] Wagner L Sweet J Butt Z Lai J Cella D Measuring patient self-reported cognitive function: development of the functional assessment of cancer therapy - cognitive function instrument J Support Oncol 2009 7 6 W32 W39

[B51] Buysse DJ Reynolds CF 3rd Monk TH Berman SR Kupfer DJ The pittsburgh sleep quality index: a new instrument for psychiatric practice and research Psychiatry Res 1989 28 2 193 213 10.1016/0165-1781(89)90047-4 2748771

[B52] Eakin EG Resnikoff PM Prewitt LM Ries AL Kaplan RM Validation of a new dyspnea measure: the UCSD shortness of breath questionnaire. University of California, San Diego Chest 1998 113 3 619 624 10.1378/chest.113.3.619 9515834

[B53] Brown WJ Trost SG Bauman A Mummery K Owen N Test-retest reliability of four physical activity measures used in population surveys J Sci Med Sport / Sports Med Aust 2004 7 2 205 215 10.1016/s1440-2440(04)80010-0 15362316

[B54] Marshall AL Miller YD Burton NW Brown WJ Measuring total and domain-specific sitting: a study of reliability and validity Med Sci Sports Exerc 2010 42 6 1094 1102 19997030 10.1249/MSS.0b013e3181c5ec18

[B55] Courneya KS Friedenreich CM Reid RD Gelmon K Mackey JR Ladha AB Proulx C Vallance JK Segal RJ Predictors of follow-up exercise behavior 6 months after a randomized trial of exercise training during breast cancer chemotherapy Breast Cancer Res Treat 2009 114 179 187 10.1007/s10549-008-9987-3 18389368

[B56] Colinet B Jacot W Bertrand D Lacombe S Bozonnat MC Daures JP Pujol JL A new simplified comorbidity score as a prognostic factor in non-small-cell lung cancer patients: description and comparison with the Charlson's index Br J Cancer 2005 93 10 1098 1105 10.1038/sj.bjc.6602836 16234816 PMC2361505

[B57] Cella D Eton DT Lai JS Peterman AH Merkel DE Combining anchor and distribution-based methods to derive minimal clinically important differences on the Functional Assessment of Cancer Therapy (FACT) anemia and fatigue scales J Pain Symptom Manage 2002 24 6 547 561 10.1016/S0885-3924(02)00529-8 12551804

[B58] National Research Council Panel on handling missing data in clinical trials. The prevention and treatment of missing data in clinical trials Committee on national statistics, division of behavioral and social sciences and education 2010 Washington DC: Pres NA

[B59] Fitzmaurice GM Laird NM Ware JH Applied longitudinal analysis 2011 2 Hoboken NJ: Wiley

[B60] White IR Horton NJ Carpenter J Pocock SJ Strategy for intention to treat analysis in randomised trials with missing outcome data BMJ 2011 342 d40 10.1136/bmj.d40 21300711 PMC3230114

[B61] Ries AL Bauldoff GS Carlin BW Casaburi R Emery CF Mahler DA Make B Rochester CL Zuwallack R Herrerias C Pulmonary rehabilitation: joint ACCP/AACVPR evidence-based clinical practice guidelines Chest 2007 131 5 Suppl 4S 42S 17494825 10.1378/chest.06-2418

